# Nowhere to go: How stigma limits the options of female drug users after release from jail

**DOI:** 10.1186/1747-597X-4-10

**Published:** 2009-05-08

**Authors:** Juliana van Olphen, Michele J Eliason, Nicholas Freudenberg, Marilyn Barnes

**Affiliations:** 1 Department of Health Education, San Francisco State University, San Francisco, CA, USA; 2 Urban Public Health Program, Hunter College, City University of New York, New York, USA; 3 Department of Administration and Interdisciplinary Studies, San Francisco State University, San Francisco, CA, USA

## Abstract

**Background:**

Drug and alcohol using women leaving prison or jail face many challenges to successful re-integration in the community and are severely hampered in their efforts by the stigma of drug or alcohol use compounded by the stigma of incarceration.

**Methods:**

This qualitative study is based on individual semi-structured interviews and focus groups with 17 women who had recently left jail about the challenges they faced on reentry.

**Results:**

Our analysis identified three major themes, which are related by the overarching influence of stigma: survival (jobs and housing), access to treatment services, and family and community reintegration.

**Conclusion:**

Stigma based on drug use and incarceration works to increase the needs of women for health and social services and at the same time, restricts their access to these services. These specific forms of stigma may amplify gender and race-based stigma. Punitive drug and social policies related to employment, housing, education, welfare, and mental health and substance abuse treatment make it extremely difficult for women to succeed.

## Background

Drug and alcohol use and abuse (hereafter referred to as drug use) have devastating effects on the lives of individuals and families, and the health of communities. In the past few decades, drug use has become heavily stigmatized, resulting in the enactment of increasingly punitive drug laws. Policies such as the federal ban on food stamps for those convicted of a drug felony and the "One Strike, You're Out" law that evicts tenants with criminal histories from public housing have disproportionately and adversely affected women, especially poor women, limiting their options for employment, housing, and education upon release. [1] Between 1980 and 2002, the U.S. jail population increased by 265% resulting in an unprecedented number of people being released from jail on a daily basis. [2] Men are still the majority of those incarcerated in correctional institutions, but since 1990 the number of incarcerated women has grown at nearly twice the rate of men. [3] Women make up an increasing proportion of jail inmates, reaching 12.7 percent of the population in 2005, compared to 10.2 percent in 1995. [4] Because of U.S. criminal justice, drug and other social policies, particularly those related to the "war on drugs," drug-related offenses account for the largest share of the increase in the number of female offenders. [5] This is especially true in California where between 1986 and 2002, the number of women in California prisons increased by 311%; [6,7] and between 1986 and 1995, drug offenses accounted for 55% of the increase in the number of women in California prisons. [8]

Rising incarceration rates have had a disproportionate impact on the health of women of color who are overrepresented in jails and prisons. [9] A Black woman is more than seven times as likely to spend time behind bars as a White woman. [10] In San Francisco County Jail (SFCJ), eight in ten women are women of color and more than half are African American, despite the fact that less than 8% of the city's population is African American. [11] Some studies have found that services within the criminal justice system may not meet the specific needs of women of color, compromising their effectiveness. [12] Incarcerated women have higher rates of health problems such as HIV, hepatitis C, [8,9,13,14] and other sexually transmitted diseases, recent and chronic substance use disorders, [15-18] and mental health problems [18,19] than the general population.

Whereas much of the public attention has focused on people entering and returning from prison, each year more than 9 million people return home from jails, facilities that house status violators, detainees awaiting adjudication, and those sentenced to less than a year. Jails are even less likely than prisons to have adequate health care, substance abuse treatment services, or vocational training. [20,21] Although the average length of stay in jail is only about 45 days, evidence suggests that even a brief incarceration disrupts the lives of individuals, families, and communities. [16] Despite the high rates of health problems among incarcerated women, few receive treatment in jail or the discharge planning or aftercare that could link them with needed services after release. [15,16] In addition, incarceration and other social policies often contribute to a downward cycle of substance use and mental health problems. For example, a brief stay in jail often results in the termination of one's benefits including Medicaid, leaving newly released inmates with reduced or delayed access to mental health services and substance use treatment. [22-24] The lapse in benefits can also mean gaps in prescription drugs, significantly affecting physical and mental health. Changes in welfare policy imposed new restrictions on women as a condition for receipt of benefits. For example, some states require women to abstain from drug use before getting benefits, [17] making it more difficult for women with substance use problems to get treatment especially given the dearth of programs to address the needs of women dependent on drugs. Incarceration can also lead to homelessness or a change in housing status, [15] thus increasing the risk of precariously housed women for victimization, exploitation, or forced return to an unsafe situation. [24] Conviction for drug felonies can negatively affect the ability to get student aid and improve one's status in life through education. [1] Finally, since incarceration is concentrated in poor communities, most people leaving jail return to communities with limited access to education, housing and jobs and high levels of poverty, racism, drugs, violence and health problems. [15,25]

### Stigma and Incarceration

Both drug use and incarceration carry stigma for men and women, [26,27] but the degree of stigma is much greater for women because of gender-based stereotypes that hold women to different standards. [28] The stigma of drug use and incarceration may be additive, yet little research to date has explored the impacts of multiple burdens of stigma on formerly incarcerated women. Stigma refers to unfavorable attitudes, beliefs, and policies directed toward people perceived to belong to an undesirable group. Erving Goffman, widely credited for conceptualizing and creating a framework for the study of stigma, described stigma as "an attribute that is deeply discrediting within a particular social interaction" (p.3). [29] Link and Phelan developed a conceptualization of stigma that describes stigmatized individuals as those who are labeled and assigned negative attributes, set apart as not fully human, and treated negatively. [30] Stigma results in prejudice and discrimination against the stigmatized group, reinforcing existing social inequalities, particularly those rooted in gender, sexuality and race. According to Link and Phelan, those who are stigmatized can experience direct, structural or internalized discrimination. For example, a formerly incarcerated woman may be treated poorly by others, denied access to housing or employment because of her criminal history, or internalize feelings of worthlessness because of the lowered expectations of those around her. This stigmatization is likely to significantly influence the success of a woman's transition from jail to home, potentially limiting her help-seeking intentions and compromising her access to health care, drug treatment, employment and housing. For many of these women, their stigma stems from the intersecting categories of incarceration history, drug use, mental health status, gender, race/ethnicity or sexual orientation, making it difficult to attribute any particular stigmatization to a single category.

Stigma contributes to policies related to the treatment of drug users, for example leading to strict standards of abstinence or clients are discharged from treatment as "failures," where victim-blaming is common as well as moral judgments about the "weak wills" of people who are thought to "choose" drug use. Incarceration stigma is expressed through a punishment rather than rehabilitation approach to drug use, a view of drug users as "criminals," zero tolerance for any use (or relapse), and a disdain for therapeutic interventions or compassion for those with drug addictions. The resultant criminalization of drug use means that relapse to drug use is the primary reason for a revocation of parole and return to prison for women. [31] The stigma can be internalized by those who use drugs as shame and guilt [27] which may exacerbate mental health problems, increase the risk for relapse, and result in low self-esteem.

In this study, we interviewed nine individuals and conducted two focus groups with eight other women who had been released from jail within the last 12 months about the challenges faced by drug users leaving jail. This report describes women's perceptions of the difficulties they faced upon release and the factors that eased their transition from jail to home.

## Methods

The purpose of this study was to better understand the experiences of women with drug problems returning home from San Francisco County Jail. Women were able to participate in the study if they had been released from jail within the last 12 months and if they had been incarcerated for offenses related to their drug use or if they reported having drug problems at the time of arrest. Nine interviews and two focus groups were conducted. Focus groups were used to supplement interviews, because many women perceive focus groups as less threatening because they reduce the power differential of researcher versus research participant. [32] IRB approval for this study was obtained from the San Francisco State University Office for the Protection of Human Subjects.

Participants were primarily recruited through public housing in the Western Addition, a predominantly African American and low-income neighborhood in San Francisco, and through Northern California Service League (NCSL), a community-based organization located near the jail that provides services including life skills training, referrals, and vocational training to formerly incarcerated individuals. Flyers about the study were posted in both locations. In addition, potential participants were identified through staff at NCSL and through one of the interviewers for this project who lived in the Western Addition. The two focus groups were held at the offices of the NCSL, and interviews were held at a time and place convenient to the participants. The focus groups and several interviews were conducted by the first author of this study and the rest of the interviews were conducted by the fourth author, who was at the time of this study an undergraduate student at San Francisco State University. Data were collected in the Fall of 2005 and Spring of 2006. The interview and focus group guides were semi-structured, with a prepared list of topics and questions related to pre- and post-release experiences, particularly related to drug use, access to housing and healthcare, employment, and family/relationship issues. However, interview and focus group participants were also encouraged to share their own stories and to engage in a meaningful conversation with the interviewer, or with other participants in the focus group and its facilitator.

Interviews were analyzed according to standard qualitative techniques. [33-35] This included assigning codes to meaningful segments of transcript text and recording memos to help make sense of the data and facilitate more abstract development of theories about the data. Based on prior research, a number of themes were identified before beginning analysis, and these themes guided the development of the questions. Themes included: pre- and post-release challenges, treatment by staff in jail and by service providers post-release, challenges finding drug treatment and health care, employment and housing discrimination, and social support. The text of the interviews and focus groups was examined for the presence of these and other emergent themes; themes that repeatedly emerged in interviews and those emphasized by the respondents as important are reported here.

## Results

### Participant characteristics

The average age of the 17 women participating in the study was 40 years (range of 22–53). The majority of participants (n = 10) were African American; the remaining seven participants described themselves as African American/White (2); White (2); Native American (1); Filipina (1); and Asian (1). Most participants (13) reported they had been in jail at least twice in the last 12 months. Most had used alcohol, heroin, methamphetamine, crack, cocaine, and/or marijuana in the past 6 months, but also tried to quit using drugs in the past six months. Most had unstable living situations, including several who were homeless or living in a car or a shelter.

### Women's experiences of stigma related to their drug use and incarceration

The themes that many women repeated can be organized into three interrelated consequences of drug and incarceration stigma: basic survival, access to needed treatment services, and family and community reintegration. These findings suggest that stigma helps to keep the revolving door of relapse, recidivism, and incarceration spinning. In the words of two respondents:

*If they don't want people going back to jail they need to build their spirits up instead of breaking their spirits down, and that's all they're doing. I mean, "You're always going to [go to jail]. You're always going to be nothing but a whore. You're always..." Ain't nothing wrong with being what I'm being if I'm not hurting anyone. I'm not a murderer. I'm not out here killing nobody. And if I sold drugs, I sure as hell wouldn't sell it to somebody if it was going to kill them. You know? I'm not crazy. I wouldn't want to live with that for the rest of my life. San Francisco needs to be more caring*.

*They [the police] say, "She's a known crack head and a crack addict." A lot of them like take my clothes and arrested me for [being] under the influence, and I'm not even high. And take my property one place and me another. Take my money. Leave my – they even don't even take my clothes. They leave my shit on the streets*.

### Survival: Jobs and housing

The first concern of most women coming out of incarceration or drug treatment is making a living. This section shares some of the respondents' experiences trying to find employment and safe housing. Several women described what they perceived as job discrimination. For example, one woman said:

When I tried to get a job, behind me being a felon, I was unable to get a lot of jobs that I applied for. Because they say be honest about your history. And you're honest. And then you end up lying anyways because you need the work. And they're not going to hire – I have been so institutionalized. Yeah, I got a great resume because it's all transferable skills. But who's going to really hire me? Who's really going to give me the opportunity to make like $50,000 a year unless I go to school for the next ten years and prove that I am like a new citizen or whatever? But in the meantime, between-time, how am I going to pay rent? You know what I mean? How I am going to take care of myself if I don't have the support?

Those who reported that they had been able to get a job felt that the jobs didn't pay them enough to survive in San Francisco or that they did not provide the benefits they needed to address their health problems. Another discussed how the cost of living in San Francisco made it even more difficult for those like her who can not find a job that provides a living wage:

*You can't get certain jobs because you're a felon. And that's what really sucks. You might have the qualifications to get this job. Just because you're a felon, I'm not going to give you this job where you can make money so you can survive in San Francisco. And that's the hard part. We have to make money to survive out here. But it's not cheap. We can't just be making nine, ten dollars an hour. It's not going to work*.

Sometimes, the challenges women faced in getting a legitimate job that pays a living wage forced them to choose between unpalatable options – for example, sex work or selling drugs:

*I'm a convicted felon, I'm not eligible for other things. Like I'm a drug addict. I'm not eligible for Proposition 36 (Appendix) because I sold dope. Well, to me, prostituting was too demeaning and I was raped too many times, so I stopped doing it. Right? So I started selling drugs. I'm still a drug addict. It's not like I sold drugs to become a rich person or anything. I sold drugs to pay my rent. I paid it. I lived in a room that was $50 a day, which was $1,500 a month*.

Many women reported that they were forced to return to unhealthy or unsafe housing environments after release from jail because they had no healthier alternatives:

*[the shelter] is drug infested... a lot of dope and it's dirty. It's scabies, body lice, hepatitis, TB, crabs, you name it*.

A few women who were able to stop using drugs after release emphasized that a safe environment post-release helped them to stop using drugs. Women searching for alternatives to homeless shelters reported that incarceration severely compromised their ability to get housing, either because of their own or their family member's drug convictions:

*Tried to go to Project Homeless Connect and everything and stuff like that to get housing, and can't get housing because my husband's got a felony. You know, stuff like that keeps us from being able to be free and have a place. All because of a drug conviction that he had, you know. It's not fair. People that are doing murders, they're getting the places that we could have*.

This particular woman said she had no intention to stay off drugs upon release because she had "nowhere to go"; she had been on a waiting list for public housing for more than three years.

### Access to Treatment Services

The majority of women reported that they had difficulty finding or benefiting from services because of the stigma of drug abuse or incarceration. Although approximately half of the women reported having been abused as children and all reported having current drug problems, few of them had received counseling or support for these problems while in jail. Whereas some women said they were offered the opportunity to participate in a jail-based drug treatment program, those who did attend said the program did not help them to address deep-seated psychological problems, often related to growing up in an unhealthy environment. One woman reported being asked to leave a program because of her behavior:

(At the program), they ask, 'Well, why are you using drugs?'

'Well, it makes me feel better.'

'Well, why does it make you feel better?'

'Okay, this is what I'm going through.'

'Well, there's no excuse.'

'No, there's no excuse but it's my choice.'

*They tell me I shouldn't talk back. Talk back? You asked me a question, I'm going to answer it... I don't expect them to kiss my ass. But what I do expect is them to be a little more compassionate and caring. People who do drugs it's because they have a misfunction in their lives... It's not because they want to hurt anybody. It's because they have a balance that's gone*.

This woman reported that the staff member's questions made her feel stigmatized as a drug user. Several other women reported that some program staff (both inside and outside the jail) made them feel worthless because of their situations and their drug use:

*That's the problem with programs. They think they're better than you because they quit using. Fine, you have a reward for that. But don't put down somebody that hasn't been able to stop yet. That's just the way I feel about it*.

However, several women reported more positive experiences in post-release programs, such as the women's group operated through the Pre-trial Diversion Program. Women are often mandated to participate in these groups as a condition of their release from jail. One woman expressed what she gained from attending this group:

*Yeah, you learn how to express yourself in different ways – culturally, mentally, physically......It helps a lot to see who is and who isn't being fairly treated. And then it helped me a lot to see that there was a lot of people like me that weren't treated very good as children. You know, that's the growing up in an unhealthy environment, so you learn there's more people that are like you*.

This woman, who had experienced childhood abuse, did not receive any help in jail for these problems. Another woman spoke about having made contact with someone from an organization serving low-income and homeless HIV-positive individuals. This staff person made a difference in her life because of the unconditional support she offered:

*And she told me – supporting me in doing the right thing wherever I was at, whether I was on drugs or I was clean and sober. She always encouraged me to do the right thing cause it's hard when you get out. You don't have no family. You don't have anyone who is really supporting you in doing the right thing because everybody expects you're going to do the wrong thing anyways, so you just go right back to what you know*.

This quote also highlights the way that the low expectations of other people in their lives led to a self-fulfilling prophecy for many women. Indeed, most women described an almost immediate return to drug use after release. Only the few women who reported that they had been released directly to a drug treatment program were exceptions to this norm. For example, one woman described how her last release was different from the previous ones:

*Previous times [I was released], cause I didn't have nowhere to go previous times. I said to myself, "Okay, now I'm going to go back outside and go back and do the same thing." Cause I have no family out here. I have nothing. I have nowhere to go, and I have no way legally, the right direction of where to go, especially the first day you get out. It's hopeless*.

This woman was eligible for Proposition 36. Another participant, however, said she was ineligible for the Proposition 36 services because she had been charged with drug selling.

For many women, the lack of treatment alternatives upon release meant an almost immediate return to their former lifestyles:

*You know, after you go back out there, you aren't even worried about the services. You don't even think about real services when you relapse... All's about here making some money. Get me a room for when I – if I get tired.... Because I'm on the block 24/7 getting some money so I can support my habit. You're not worried about getting benefits anybody has*.

*Well, for me the last time that I had got out it was difficult even though I had an exit plan. I still was homeless. The Exit Plan was – it sounded good, and it looked good on paper. But the reality of it was when I came home, I – I mean the place where I was living was a crack house, and I didn't want to go back to the environment. But being that I came out with no ID, nothing, no money, nothing, I went right back to selling dope. I went right back to my drug addiction... I ended up back in jail two weeks later behind my drug addictions*.

This second quote illustrates that an exit plan (a plan detailing what someone will do upon release to ensure successful reintegration), without the stable supports of an income or a drug-free place to live, may carry little benefit to someone leaving jail. Even those women who acknowledged the importance of the internal motivation and desire to quit admitted that these good intentions were not sufficient in the absence of a safe environment:

*[Getting off drugs] got to be something that they want. Because I know I've had good intentions 101 times getting out, and I swore that I was not going to get high. And I always ended up loaded because I didn't have a safe place to go to. I went right back to the same environment and I kept all the same friends. And it's like people, places and things. And it's all triggers. And it's just all bad. And if you know nothing but drug addicts, you're going to go and do drugs. It's just like second nature. You know, to me, it's like I know how to hustle and get money. I don't know how to get a job. I just barely learned how to fill out a application. So it's things like that*.

For this woman, drugs were the only way of life that she knew and living without drugs would mean learning a whole new way of living. When asked what they did in the first 24 hours after release, most women reported immediately returning to familiar places and people and getting high:

*When you get the fresh air (after release), you lose your mind anyway. It's like freedom, and then you go see this man and this woman or whoever it is, you know, you get caught up in the codependency of drug addiction, and you want to go and have sex or whatever you haven't done in a mighty long time. You want to go and do that. So I think it would be helpful...if you could come and get (me) when I get discharged*.

Other women told similar stories about the pervasiveness of addiction.

Participant comments about the challenges of gaining access to city services revealed both structural barriers such as lack of coordination among agencies and individual barriers related to their drug dependency. One woman with mental health problems talked about being unable to be housed in a shelter and being endlessly shuffled around the city, referred from one place to another because of her mental health problems. This woman said she was living under the freeways.

*So until I can learn how to deal my emotions, they feel it's better for me not to be in a shelter. So I was diagnosed [with borderline personality disorder] and I'm waiting – I'm on SSI and pending right now. I'm waiting. Waiting. Waiting. I don't know what's going to happen, but I want to get the counseling. And I want to get the help I need. And just I'm still waiting. Referral here. Referral there. Instead of actions, they're just referring me and referring and referring me. And it's getting aggravating*.

### Family and community reintegration

Three respondents expressed a deep desire to reunite with their children, arguing that the need to be good role models for their children was what compelled them to quit using drugs.

*When I came home, [my daughter] was, almost two years old. And she came over to me and put her hand on me and looked at me and said, "Mommy, I love you." And the fight was on from there. What's helping me now to abstain – I don't care how I look because I'm really abstaining*.

Those who expressed a desire to reunite with their children also said they lacked a safe place where they could live with their children. The predominant experience of respondents who were mothers was that their continued substance use was a significant barrier to reunification. Several women felt they needed to be given more choices, rather than be pressured to reunite with their children when they may not be ready to do so:

*You have to sit down and you have to find out what her plan is and what she feels. And if the other people around her are telling her, 'You have to do this or you don't love your kid,' that's going to make it even worse...as long as she's in a forced situation... and you don't know which way to turn, and you don't know what to do, and if you fuck up this way, you're going to lose your kid permanently. And that's usually what happens*.

Two women talked about giving up their children because they wanted the best for their offspring, as illustrated by the following quotes:

*So I couldn't put him through that (feeling of abandonment). So when he asked me to stay (with his foster parents), I let him stay. I signed him over to them. I just couldn't – it's not that I don't love my kids. It's just that I felt they'd be better off where they were*.

*My son is fourteen, and I have two daughters, an eight-year-old and a five-year-old. In their whole lives, I've been incarcerated, in and out of their lives. I don't even know that it would be helping to even be a part of their lives right now because what if I do relapse? What if I can't get it together? I mean I'm so used to failing, it's like to believe that I can actually succeed, I might set myself up and fail first before I make it to the miracle*.

These quotes reveal the difficult choices some women are forced to make in the face of the challenges they faced getting a job, housing, and recovering from drug use to achieve enough stability to become good mothers to their children. They also noted that there is little support to get clean and sober in their home communities.

*Because everybody I know is stuck in the same situation, not nothing positive. It's always crack, nothing regards achieving or talking about a goal or what do they want in life. But it's always crack. When I feel down and out and depressed, and I be trying to talk about my problems, but they be acting like they hear but they talking about a pipe*.

Many participants discussed the cumulative stress and social isolation caused by incarceration that ultimately disrupted family and community connections.

*And when I was in jail, it was just like they were taking little pieces of me away. And it's like if I stayed there longer, I probably would have been just totaled. You know, just totaled. I don't know if I could have handled it. I mean, I've done six months. Nobody visiting me. Nobody giving me any money. Nothing. I was there, and it was hard. It was really hard*.

*When I went to [jail], a lot of shit happened. My brother got his throat cut. My niece, she took a pill and almost OD'd. When I got out of the pen, when I came home, I knew my grandmother was dying. It was a lot of things, my family, there was no unity*.

### Suggestions generated by the respondents

Some of the women offered suggestions for improving the plight of drug users coming out of jail. For example, some mentioned the need for meaningful discharge planning, and another woman, quoted below, suggested peer education.

*I think that if we were to help one another in the recidivism of our own lives is to become like – be peer educators and learn how to do some type of intervention for women who are transitioning... so we start this process 30 days to your discharge date and learn, okay, you get the people who like really want to change and do something different*.

Some women felt strongly that they needed sober housing, for example *: "a safe environment, alcohol and drugs and toxic-free. When I say toxic, I mean [free] of the negativity." *Yet other women stressed the importance of housing for women who are still using, illustrating the heterogeneity of the population:

*I just don't feel that (being abstinent) should be part of the rules. A lot of people will want to get off, but still use while they're going. And will go and slowly be tired of it, because you're not going to get off drugs until you're tired of it. You're not – you can't get off because you're forced to. Because if you do, you're going to relapse. You're going to relapse hard*.

One participant shared a positive experience with early release from jail to a program under Proposition 36:

*Previous times (I was released) I didn't have nowhere to go. I said to myself, "Okay, now I'm going to go back outside and go back and do the same thing... Prop. 36 they put me into a program. It's transitional. It helped me out a lot. It gave me housing and stability. And I'm happy*.

Some women reported participating in the San Francisco Pretrial Diversion Program where they were mandated to go to groups as a condition of their release from jail. Benefits of the groups included

...*learning how to express yourself in different ways*.

*It helped me a lot to see that there was a lot of people like me that weren't treated very good as children*.

## Discussion

This study has several limitations. This was a convenience sample of women, who may differ from the general population of women released from San Francisco County Jail in significant ways. In addition, the small sample size did not allow comparisons in perspectives on reentry by race/ethnicity or other personal characteristics. Since this study was designed to better understand women's reentry experiences in San Francisco, it may not reflect the experiences of women in other jurisdictions who are returning to their communities. In addition, it is important to note that this study was designed to elicit women's pre-release and post-release experiences and the challenges they experienced trying to find city programs and services. This study did not attempt to ascertain the veracity of women's self-reports.

A central theme throughout women's narratives revolved around the double stigma of being a drug user and having a history of incarceration. Drug users or those involved in the illicit drug economy are considered "suspect populations," groups of individuals who are highly stigmatized. [36] Public response to such populations, including public policy, attempt to contain and manage these individuals by stigmatizing drug use as a deterrent strategy. [37] Indeed, laws enacted as part of the nationwide War on Drugs have resulted in harsh penalties for drug users, including the prohibition of people with drug-related felonies from getting government assistance such as public housing and federal financial aid to attend college. In most cases, violent felons still have access to these benefits. [38] Finding employment after release was also exacerbated by incarceration-related stigma. Even those with transferable job skills said that employers were unwilling to hire them as soon as they learned of their criminal history. Some suggested they were better off lying to prospective employers about their criminal history, perhaps an important survival strategy given research that has shown that 65% of employers would not knowingly hire someone who is formerly incarcerated, regardless of the offense. [27,39]

Although all of the women who participated in this study had recently been released from jail, not prison, the stories they told demonstrated ways that even a short stay in jail can disrupt one's life. The participants faced multiple, interrelated problems after release from jail, including drug use and mental health problems, family problems, lack of safe housing or a job that pays a living wage, challenges finding needed services, and social isolation, all stemming at least in part from stigma and discrimination related to their drug use and history of incarceration. Most of the women left jail unprepared to meet these challenges, some of which are related to regulations and practices that have inadvertently made successful community reentry more challenging. Few of the women we spoke to had received pre-release planning that helped them to find stable housing, find services or a job, or reunite with family members. Even women (usually only those who participate in programs in jail) who received an exit plan found that the plan was of little practical use on the outside. Pre-release planning can be a challenge when a woman's length of stay in jail is unknown or may last only a few days, but if reentry planning begins at a person's point of entry into the criminal justice system it has potential to reduce substance use and improve community health and public safety. Prior to release, women should be linked with community service providers who can help address their needs (e.g., for housing, employment etc.) after release.

Policies also hindered women's successful reintegration after release. For example, housing policies [40,41] often made it more difficult for people returning from jail to find stable housing. Lack of options after release, particularly a safe place to live, for most women we spoke to propelled them back into criminal activity, most often including drug use and drug selling as a survival mechanism. In the face of limited options, including a lack of gender-specific programs that allow women to live with their children or that provide childcare services [42-44] many women may be forced to choose between continuing custody and care for their children and drug treatment. The complexity of women's lives after release suggests that interventions designed to facilitate successful community reintegration must provide a range of services including substance use and mental health treatment, outreach, behavioral skills groups, intensive case management, reproductive health services, and programs linking women to housing and job training. Ironically, the social conditions that impaired their re-integration after a jail stay were typically the same conditions that led to their drug use and incarceration in the first place.

Although the mental health status of all of the women participating was not known, the evidence presented here suggests that many were struggling with mental health problems in addition to substance use problems. Other investigators have found that the dually diagnosed are a distinct population, in need of specialized integrated substance abuse and mental health treatment. [45] Stigma may be both a consequence of mental health status and a contributing factor to mental health problems because internalized shame creates emotional distress.

In San Francisco, Oakland and in other cities nationwide, a successful campaign known as "Ban the Box" has resulted in the elimination of the box on a form that applicants are required to check if they have had a felony conviction in the past. [46] While employers may still check the criminal records of prospective employees, the elimination of the box levels the playing field so applicants with a criminal history are at least initially considered alongside the other applicants in the pool. More efforts such as these are needed to combat the systematic social exclusion that results from the stigmatization of drug users who become entangled with the criminal justice system.

Finally, women's stories clearly illustrated how they felt poorly treated because of their status as a drug user, and the challenges women face finding the services they need are compounded by the quality of their interactions with service providers. For many women, the perception that they were being treated as an inferior exacerbated emotional problems with which women already struggled and contributed to relapse to drug use. [47] Stigma also resulted in mistaken assumptions about a woman's needs or priorities during reentry. While reunification with children might be a priority for some women, some who shared their stories clearly did not want to be pressured to make an immediate choice that might not be right for them or their children. Future research should more systematically examine the ways in which stigma exacerbates the problems women face after release, and constrains the opportunities available to them. There is also a need for studies to investigate how stigma might be internalized, exacerbating mental health problems and contributing to drug relapse.

Finally, incarceration and drug use added to the burden of stigma already elicited by the gender and race/ethnicity of the participants in this study, categories with lower levels of power in our society. In addition, gender and race/ethnicity play a major role in shaping the opportunities that women drug users leaving jail face, demonstrating the importance of understanding the intersecting patterns of stigma that block women leaving jail from successful reentry.

## Conclusion

In an effort to discourage drug use and reduce crime, elected officials instituted policies intended to punish and stigmatize drug users to serve as a deterrent to drug use. This report showed that for the women who are the actual targets of these policies, the real impact was often the reverse: punitive policies, lack of services and stigmatization encouraged a return to drug use, increased criminal activity and re-incarceration, and exacerbated individual and community health problems. Even when women were able to find services, the stigma of drug use and incarceration often affected the quality of the interactions, even with providers of therapeutic services. In the future, launching campaigns to reduce the intersecting stigmas of drug use, incarceration, gender, and race/ethnicity, drawing on strategies currently used to improve mental health outcomes [48,49] may enhance the effectiveness of reintegration services while also assisting women leaving jail to find the support they need for successful reintegration into their families and communities.

## Competing interests

The authors declare that they have no competing interests.

## Authors' contributions

JVO conceived of the project and carried it out, ME assisted with organization and interpretation of the data, NF provided guidance on study design, data analysis, interpretation and organization of the manuscript, and MB assisted with conceptualization and implementation of the project, conducted some of the interviews, and reviewed the paper.

## Appendix

Proposition 36, the Substance Abuse and Crime Prevention Law act, was passed in California in 2000. It allows first- and second-time nonviolent offenders charged with drug possession the opportunity to receive substance abuse treatment instead of incarceration.
